# Direct comparison of supervised and semi-supervised retraining approaches for co-adaptive BCIs

**DOI:** 10.1007/s11517-019-02047-1

**Published:** 2019-09-14

**Authors:** Andreas Schwarz, Julia Brandstetter, Joana Pereira, Gernot R. Müller-Putz

**Affiliations:** Institute of Neural Engineering, University of Technology, Stremayrgasse 16/IV, Graz, 8010 Austria

**Keywords:** Brain–computer interface (BCI), Motor imagery, Co-adaptive BCI, Semi-supervised learning, Supervised learning

## Abstract

For Brain-Computer interfaces (BCIs), system calibration is a lengthy but necessary process for successful operation. Co-adaptive BCIs aim to shorten training and imply positive motivation to users by presenting feedback already at early stages: After just 5 min of gathering calibration data, the systems are able to provide feedback and engage users in a mutual learning process. In this work, we investigate whether the retraining stage of co-adaptive BCIs can be adapted to a semi-supervised concept, where only a small amount of labeled data is available and all additional data needs to be labeled by the BCI itself. The aim of the current work was to evaluate whether a semi-supervised co-adaptive BCI could successfully compete with a supervised co-adaptive BCI model. In a supporting two-class (190 trials per condition) BCI study based on motor imagery tasks, we evaluated both approaches in two separate groups of 10 participants online, while we simulated the other approach in each group offline. Our results indicate that despite the lack of true labeled data, the semi-supervised driven BCI did not perform significantly worse (*p* > 0.05) than the supervised counterpart. We believe that these findings contribute to developing BCIs for long-term use, where continuous adaptation becomes imperative for maintaining meaningful BCI performance.

**Electronic supplementary material:**

The online version of this article (10.1007/s11517-019-02047-1) contains supplementary material, which is available to authorized users.

**Graphical abstract:**
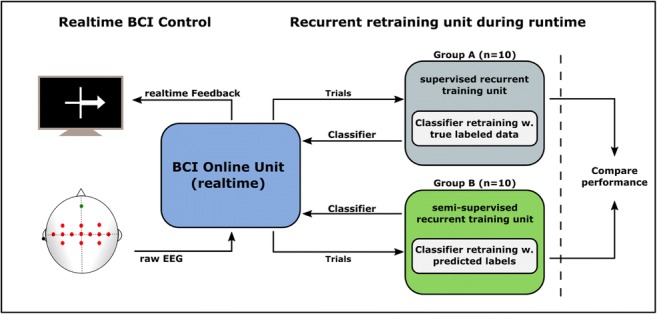
In this work, we investigate whether the retraining stage of a co-adaptive BCI can be adapted to a semi-supervised concept, where only a small amount of labeled data is available and all additional data needs to be labeled by the BCI itself. In two groups of 10 persons, we evaluate a supervised as well as a semi-supervised approach. Our results indicate that despite the lack of true labeled data, the semi-supervised driven BCI did not perform significantly worse (*p* > 0.05) than the supervised counterpart.

## Introduction

Brain–computer interfaces (BCIs) have a wide range of potential applications, whether in rehabilitation after stroke [1–3], restoring lost motor functions due to spinal cord injury [4–6], or in acquiring deeper understanding of brain functionality [7, 8]. One of the main brain activity patterns used to design EEG-based BCIs is event-related desynchronization/synchronization (ERD/S) [9]. ERD/S can be elicited during several mental tasks [10], being one of the most common tasks the repeated mental imagery (MI) of a movement task. One example [9] is squeezing a stress ball or plantar flexion/extension of both feet. These MI tasks lead to ERD in the alpha (8–12 Hz) and beta (16–24 Hz) frequency bands during the task, and beta ERS after movement.

In order to operate an MI-based BCI, it is typically necessary to perform a calibration step, which can be a tedious task. In this calibration step, usually dozens of repetitions for each condition are recorded to provide enough training data for preprocessing and classification algorithms [11–14], while the user does not receive any feedback. Once calibrated and operable, the BCI attempts to decode the mental imagery of the user based on its initial calibration data set, assuming stationarity of the incoming EEG signals. Hence, this type of BCI is not taking into account that EEG measurements are inherently non-stationary since the brain processes vary over time and are particularly affected by user fatigue, loss of attention, and even learning processes [15, 16] during the execution of the task. Aside from that, also changes in electrode impedances or other external factors can affect in real time the performance of these systems. Based on the work of Vidaurre et al. [17, 18], Faller et al. [19] developed a co-adaptive training procedure, where feedback to users was provided almost instantly: After a short data collection phase (10 trials per condition (TPC)), the system was trained and already provided feedback to users based on their actions. On recurrent training steps, the system retrained with all available data to improve on feedback performance. In this way, users and the BCI were engaged in a setup of mutual learning. We further developed the approach in [20] by integrating state-of-the-art machine learning methods and could show in an identical experimental setup that our changes lead to a significant improvement in BCI performance by 13% on average.

Both studies could show the feasibility of early feedback presentation. Moreover, these co-adaptive systems acknowledged BCI operating users as “learners” and engaged them in a supervised mutual learning environment.

Nevertheless, these systems still rely on accurate class label information to perform model updates to effectively counter non-stationarity effects of the EEG. In a scenario of long-term, self-paced BCI use, where users do not follow a predetermined set of instructions (trials) rather than follow their causal user intention, this class label information might not be available anymore.

One approach to combine adaptive BCIs with this real-life setting is the use of unsupervised learning in adapting the classifier, which has been proposed in several studies: Hasan et al. [21] suggested an unsupervised Gaussian mixture model, which they incrementally updated in offline testing and tuning model parameters to optimally combine older and new data. Their results showed that the unsupervised adaptation performs better than no adaptation, but still worse than a classic supervised approach. Gan et al. [22] and Vidaurre et al. [23] developed methods for unsupervised adaptation of LDA classifiers by updating their mean and variance. In Gan’s paper [22], the BCI system was constantly adapted after every trial by means of unsupervised clustering and subsequent updating of the LDA’s mean and variance. In an online simulation of three subjects, this method proved to be effective. Vidaurre et al. [23] successfully adapted the LDA’s bias in an unsupervised manner which showed no difference to adaptation using class labels in an online study. All approaches attempt to improve BCI performance by counteracting the non-stationarities of EEG data in long-term BCI use. Other solutions involve stopping the recurrent adaptation [24] or provide no real-time adaptation at all [25, 26].

Our idea to tackle this issue involves the concept of semi-supervised learning: Initial calibration is performed on a comparable small set of true labeled data, but after a predefined point, only the output of the classification model is used to generate artificial labels for future data to perform recurrent trainings. Several studies have used semi-supervised learning in BCI systems, whether for a P300-based BCI speller system [27, 28] or ERD-based BCIs approaches [29–31].

In our study, we apply on the co-adaptive online BCI model presented in [19, 20, 32] the concept of a semi-supervised retraining unit. After initial supervised calibration incorporating 50 TPC (two classes), this unit performs recurrent retrainings only using artificially generated labels. The idea is to combine the best of two worlds: On the one hand, the co-adaptive approach ensures that feedback to the user is presented already after a short period of time and engages the user in a mutual learning process with the BCI. The recurrent trainings attenuate non-stationary properties of the EEG. The semi-supervised concept, on the other hand, enables the BCI to become independent of labeled data—which is imperative for operating the BCI in a truly non-predetermined, self-paced manner.

We tested this novel approach in an extended two-class MI online BCI experiment incorporating 10 healthy participants, who performed in total 190 trials per class. Additionally, we introduced three major breaks to allow potential EEG non-stationarities to take effect. Following the same experimental setup, we compare the semi-supervised concept with the supervised co-adaptive approach described in Schwarz et al. [20] on a second group of participants (*n* = 10) for comparison: We hypothesize that the semi-supervised co-adaptive approach does not perform significantly worse than the supervised co-adaptive approach, in which the classification model is trained with known true labels. We validate our hypothesis in an offline scenario where we apply each counter approach on the designated participant population.

## Methods

### Participants

Twenty healthy volunteers participated in this study. They were without any known medical conditions, and had normal or corrected to normal vision. At the beginning of the study, they were briefed about the aims of the study and gave written informed consent to participate. The study was conducted in accordance with the guidelines for ethical research according to the Declaration of Helsinki.

We assigned all participants in two groups of 10 by random selection. While group A performed the *online* experiment using the co-adaptive BCI with supervised recurrent training, similar as in [20], group B performed the *online* experiment using a co-adaptive BCI with semi-supervised recurrent training.

### Data acquisition

**Fig. 1:**
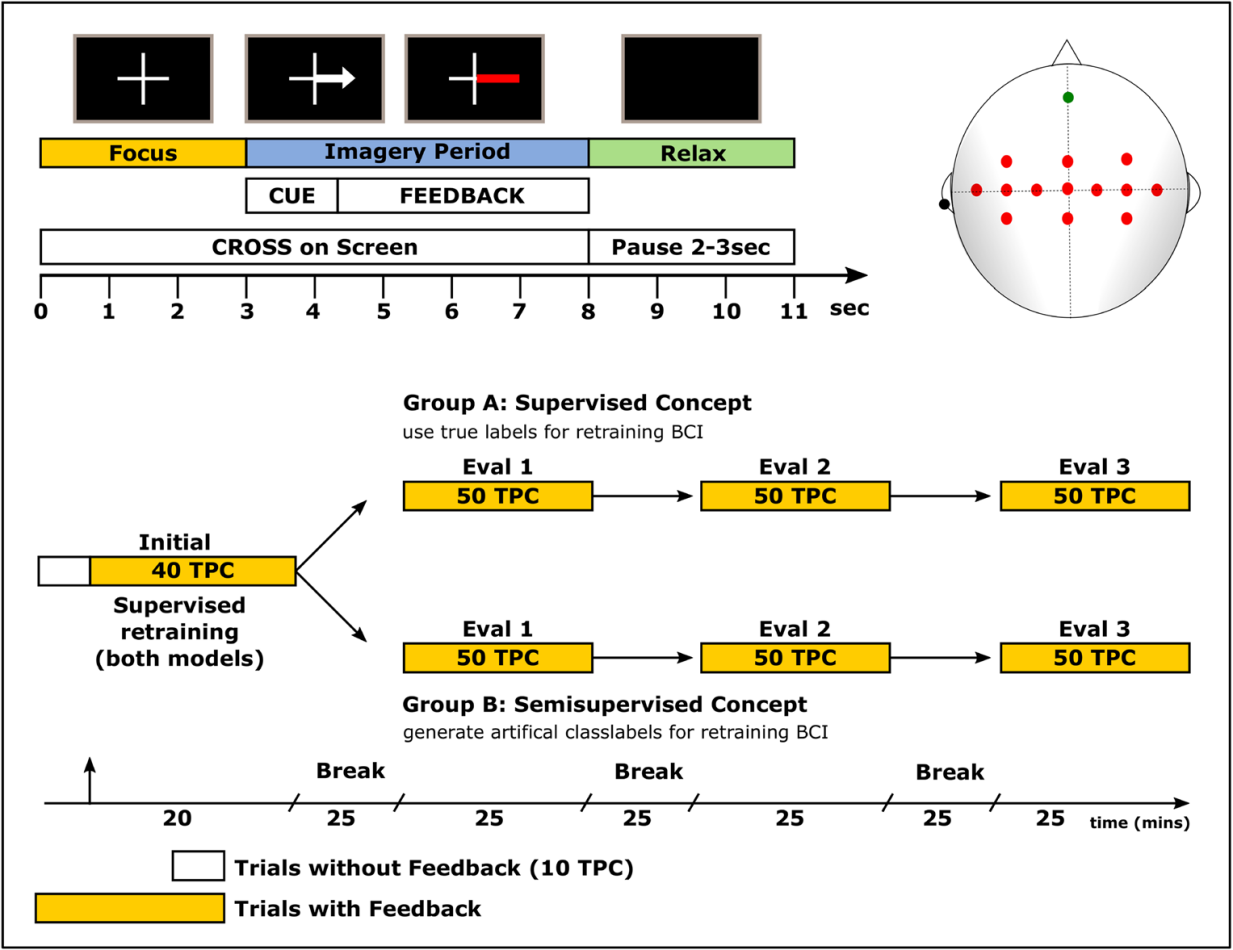
Experimental setup, Paradigm and electrode layout. *Top left*: paradigm. At second 0, a cross appeared on the screen. At second 3, the cue is presented followed by a 5-s imagery period. *Top right*: electrode setup. Thirteen electrodes (in red) were used covering the motor cortex. Ground was positioned at AFz (green electrode) and for the reference we used an electrode clip at the left earlobe. **Bottom:** Experimental timeline. The first 10 TPC were done without feedback for gathering data to allow initial classifier calibration. Thereafter, participants received feedback according to their actions. For the first 40, TPC model calibration was performed incorporating the supervised retraining unit (Initial). Thereafter, we changed the retraining concept for group B to the semi-supervised retraining unit, while we kept the supervised retraining concept for group A.

We measured EEG with 13 active electrodes (see Fig. 1, right) covering locations over the motor cortex (FC3, FCz, FC4, C5, C3, C1, Cz, C2, C4, C6, CP3, CPz, CP4). The reference electrode was placed on the left earlobe, ground on position AFz. We recorded the signals using a biosignal amplifier (g.USBamp) and a g.GAMMAsys/g.LADYbird active electrode system (g.tec medical engineering GmbH, Graz, Austria). Signals were sampled at 256 Hz and band-pass filtered from 0.01 Hz to 100 Hz (8th-order Chebyshev filter). Power line interference was suppressed with a notch filter at 50 Hz.

### Paradigm, experimental setup, and user feedback

All recordings were conducted at the BCI-Lab of the Institute of Neural Engineering at the Graz University of Technology. Participants were seated in an electromagnetic and noise shielded room to facilitate consistent measurement conditions. Instructions were shown on a screen positioned at about 1 m in front of the participants. The paradigm was based on the standard Graz-BCI paradigm as shown in [7] (see Fig. 1, top left). At second 0, a cross was shown on the screen. After 3 s, an arrow pointing downwards or to the right appeared on the screen and participants were asked to imagine for 5 s repeated plantar flexion and extension of both feet (arrow pointing downwards) or repeated squeezing of a stress ball (arrow pointing to the right), respectively.

In the breaks (~ 25 min) between the four experimental blocks, participants relaxed and watched a documentary and a TV series.

In trials in which participants were already receiving feedback according to their actions, a bar was shown at 1.25 s within the imagination period.

In both groups, participants performed 190 trials per class (TPC) each in 19 runs (10 TPC per run) whereas the first 10 trials were performed without feedback. After these first 10 trials and for both groups, we performed the first recurrent training and started to give feedback based on the user’s actions. Feedback was presented in the form of a bar. Its length was calculated by the amount of correct classifications during the last second. If less than 50% of these classifications were correct, we showed no feedback at all since we wanted to facilitate only positive feedback [33]. We updated the feedback bar 16 times per second.

We introduced three extended breaks of about 25 min in our experimental design after 40, 90, and 140 TPC (see Fig. 1, bottom). Participants remained seated in front of the display and watched a nature documentary (breaks 1 and 2) and an episode of a popular TV series (break 3).

### Online BCI experiments

The BCI system was implemented in MATLAB/Simulink (Mathworks, Natick, MA, USA) and consisted of two main units: the online unit and the recurrent training unit. The online unit processed and classified incoming EEG data and generated the feedback in real time. In parallel, EEG data was processed by the recurrent training unit, which performed recurrent updates in the inter-trial intervals. For both participant groups, the online unit was identical, whereas the recurrent training unit performed updates incorporating a supervised concept (group A) or applied the proposed semi-supervised concept (group B).

#### Online unit

We preprocessed the EEG using a filterbank [34] incorporating 15 IIR bandpass filters of order 8. Filters were built overlapping in the mu and beta range (μ-band = 6–8, 7–9, 8–10, 9–11, 10–12, 11–13, 12–14 Hz; β-band = 14–19, 17–22, 20–25, 23–28, 26–31, 29–34, 32–37, 35–40 Hz). We combined each filter with a shrinkage regularized common spatial patterns filter (CSP) to maximize class discriminability [12]. From the resulting projections, we used the first three and the last three (CSP) projections (which contain the most discriminable information for both conditions) for further processing. Subsequently, we calculated for each remaining CSP projection (6 per filter) the signal band power using a window of 1 s and applied the logarithm to ensure normal distribution. In this way, we extracted 90 features for classification (15 (filters) × 6 (bandpower features extracted from CSP projections)).

These features were classified incorporating a shrinkage regularized based linear discriminant analysis (sLDA) classifier [11, 35, 36]. The sLDA output was then used to adapt the length of the feedback bar as described in Section 2.3; however, for the first 10 TPC in the experiment, participants did not receive feedback since the first retraining was done after this period. After each completed trial, its prefiltered EEG correlate as well as lda scores were transferred to the designated recurrent training model.

#### Supervised recurrent training unit (group A)

The supervised recurrent training unit operated in parallel to the online unit and gathered all available EEG data in real time. As soon as the necessary amount of trials for retraining was accumulated (10 TPC for first training, 5 new TPC for recurrent trainings), a statistical outlier rejection was applied to all available data [19]. Here, we filtered the EEG between 3 and 35 Hz and rejected contaminated trials based on amplitude threshold (amplitude values exceeding ± 125 μV), abnormal joint probability, and abnormal kurtosis. The threshold was set to four times the standard deviation for the latter two. If five new trials passed this procedure, we accumulated them in the training datapool and performed retraining for both CSP filters and the sLDA classification model: For retraining the CSP filters, we extracted from each prefiltered trial an epoch of 3 s from 4.75 to 7.75 s with respect to the trial start [12, 37]. The sLDA classification model was trained on features extracted 5.5 s after trial start. Once the new models were calculated, the Online Unit was updated in the next available inter-trial interval. A detailed diagram of the supervised recurrent training unit can be found in the supplementary material Fig. S1. In the supervised approach, all true class labels were continuously used in the recurrent trainings of the system.

#### Semi-supervised recurrent training unit (group B)

Analogous to the supervised mode, the semi-supervised recurrent training unit assembled all available trials and continuously checked the amount of accumulated training data. Whenever five new trials per condition passed the outlier rejection, a retraining was initiated. For the first 40 TPC collected, updates happened in supervised mode, using true class labels for the training of CSP filters and the sLDA classifier (see above).

After this first calibration period, we introduced one additional selection criterion: Whenever five new TPC had passed the outlier rejection to initiate an update (accumulation of five new TPC), they were filtered according to their class probabilities. For this, we calculated for each data point within the feedback period its LDA linear score value. Thereafter, we applied the standard exponential function on each value (*z*) and normalized it by the sum of all exponentials (see eq. 1). This so-called softmax function ensures that each component is mapped in the interval (0,1), and its components add up to 1 and thus can be interpreted as (class) probabilities [38].

Softmax transformation. *K* is the number of classes and *z* the vector of the linear scores per classified sample

1 $$ \sigma {(z)}_i=\frac{\ {e}^{z_i}}{\sum_{j=1}^K{e}^{z_j}}{R}^K\to {R}^K\kern0.5em \mathrm{for}\ i=1\dots K\ \mathrm{and}\ z=\left({z}_1\dots {z}_K\right)\ \epsilon\ {R}^K $$

For each trial, we then selected the peak class probability and calculated the first (25%) and the third (75%) quartile. For further retraining, we discarded all trials whose class probabilities were outside these borders and used the remaining trials for recalculating CSP filters and sLDA classification model similar as in the supervised approach.

While this additional criterion led actually to fewer retrainings of the overall system, it ensured that apart from the initial 40 TPC, the CSP and sLDA trainings utilized exclusively the system’s predicted class labels. A detailed diagram of the semi-supervised recurrent training unit can be found in the supplementary material Fig. S2.

### Offline analysis

In addition to the online BCI experiments, we also recreated both models offline and used the data of both groups for evaluation: for group A, in which the supervised retraining approach was applied online, we reenacted the semi-supervised retraining approach offline; for group B (semi-supervised online), we reenacted the supervised approach.

Furthermore, we performed power analysis in the frequency domain using the power spectral density (PSD) estimate. For each participant, we calculated the Laplacian derivations [39] of channels C3, Cz, and C4 and used a Welch window (1 s length, no overlap) over the feedback period of each trial. Thereafter, we calculated the mean over all trials for each condition.

### Results

**Fig. 2:**
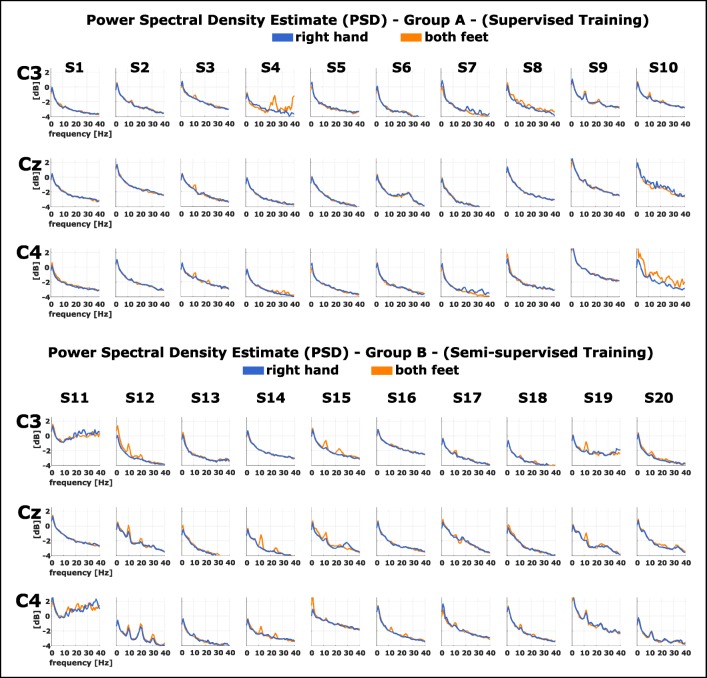
Power spectral density (PSD) estimate per subject for channels C3, Cz, and C4. Abscissa shows the frequency (Hz) while the ordinate axis reflects the power (dB)

Figure 2 shows PSD estimates for each subject of both groups participating in the experiment. We calculated the Laplacian derivations of channels C3, Cz, and C4 and used a Welch window (1 s length, no overlap) over the feedback period of each trial to calculate the PSD. The plots show the averages per subject per condition (right hand vs. both feet).

**Fig. 3:**
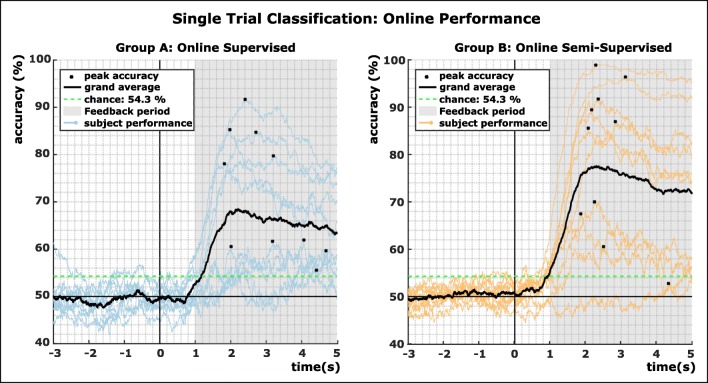
Online accuracies of the single trial classification of both groups. A total of 180 feedback trials per condition were evaluated. Second 0 represent the cue onset. Colored lines show the subject-specific performance results, the black line the grand average over all subjects

Figure 3 depicts the online performance of both groups. We evaluated all 180 feedback trials for each subject. In general, participants of group A (supervised model) achieved lower classification performance in terms of peak accuracy (average 71.9% vs. 80.0%) as well as mean accuracy (64.06% vs. 72.7%, calculated over the feedback period from second 4 to second 8) than group B (semi-supervised model). However, the performance of one participant of group B was at chance level. We used and adjusted Wald interval to determine the chance level which takes into consideration the number of trials recorded, and lies at 54.3% (alpha = 0.05) [40, 41].

Results of two-sided *t* tests calculated over all participants’ mean and peak accuracies of both groups show no significant difference (mean, *p* = 0.1931; peak, *p* = 0.2293).

We also evaluated the classification performance of both groups separately for the calibration phase (Initial 40 TPC), the first break (Eval 1, 50 TPC), the second break (Eval 2, 50 TPC), and after the last break (Eval 3, 50 TPC) (see Table 1). We conducted two one-way repeated measures ANOVA (one for each group) to determine whether there are significant differences in peak or mean accuracies between the different phases of the experiment. Mauchly’s test for sphericity indicated that the assumption of sphericity had been violated, and therefore a correction factor to the degrees of freedom of the F-distribution was computed using the Greenhouse–Geisser criterion. No statistically significant differences in mean (supervised, *F*_3,27_ = 3.165, *p* > 0.05; semi-supervised, *F*_3,27_ = 0.731, *p* > 0.05) or peak accuracies (supervised, *F*_3,27_ = 2.027, *p* > 0.05; semi-supervised, *F*_3,27_ = 0.635, *p* > 0.05) could be found for both groups. Lastly, we confirmed that there were no significant differences of mean and peak accuracies between all stages of both approaches using a mixed ANOVA (mean, *F*_3,54_ = 2.058, *p* > 0.05; peak, *F*_3,54_ = 1.220, *p* > 0.05).

**Table 1:** Intermediate performance evaluation of all 4 stages of the experiment (no significant difference between stages could be found within an approach and over all stages of both approaches)

**Stage**	**Supervised**		**Semi-supervised**	
	Peak (%)	Mean (%)	Peak (%)	Mean (%)
Initial (1–40 TPC)	70.5	60.0	82.2	74.3
Eval 1 (41–90 TPC)	76.7	66.5	83.1	75.3
Eval 2 (91–140 TPC)	77.0	68.5	83.6	75.8
Eval 3 (141–180 TPC)	75.6	66.9	81.2	73.2

**Fig. 4:**
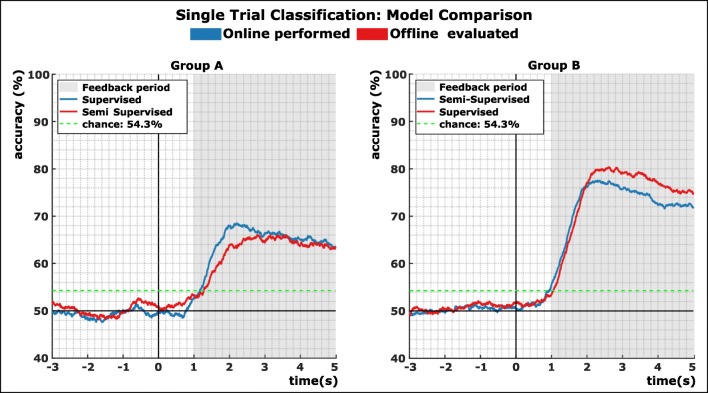
Model comparison within group. The blue lines show the grand average of all subjects performed in the online part of the experiment. The red lines show the grand average of the offline simulation results of the corresponding model

Figure 4 shows the grand average for both groups of the online performance as well as the offline simulation of the counter model. The left plot shows both averages as well as the points of peak accuracy for group A. Here, the supervised approach was performed online, while we simulated the semi-supervised approach offline. The course of both curves is similar, and no statistical differences in peak and mean accuracies were found (calculated over the feedback period from second 4 to second 8; Wilcoxon rank sum (WRS) test, *Z*_peak_ = 0.97, *Z*_mean_ = 0.52, *p* > 0.05). For group B (Fig. 4, right), the semi-supervised approach was performed online whereas the supervised approach was simulated offline. Again no significant differences of peak and mean accuracies were found (WRS, *Z*_peak_ = 0.79, *Z*_mean_ = 0.79, *p* > 0.05). In both approaches, whether performed online or simulated offline, the supervised model performed on average better by 2%.

Table 2 shows the number of recurrent trainings per approach for each subject. For the supervised approach, recurrent trainings happened considerably more often since the criterion for retraining was less strict. Finally, Table 3 shows the peak and mean accuracies for all subjects for both approaches online and offline.

**Table 2:** Number of recurrent retrainings performed per subject

	**Group A: supervised training**			**Group B: semi-supervised training**	
**Number**	**Online**	**Offline** **Semi-supervised**	**Number**	**Online**	**Offline** **Supervised**
S1	27	8	S11	7	23
S2	29	8	S12	15	29
S3	31	10	S13	13	31
S4	29	8	S14	10	32
S5	29	9	S15	18	32
S6	22	7	S16	8	27
S7	27	9	S17	11	28
S8	25	12	S18	13	27
S9	28	11	S19	12	26
S10	23	8	S20	7	32

For S1–S10 (group A), we simulated the semi-supervised approach offline, while we simulated the supervised approach for S11–S19 (group B). Due to the more strict selection criterion of the semi-supervised group, the number of retrainings performed in this group was considerably lower

**Table 3:** Online and offline performance for both groups (mean accuracy was calculated over the feedback period from second 4 to second 8)

	**Group A**					**Group B**			
**Number**	**Online** **Supervised**		**Offline** **Semi-supervised**		**Number**	**Online** **Semi-supervised**		**Offline** **Supervised**	
	Peak (%)	Mean (%)	Peak (%)	Mean (%)		Peak (%)	Mean (%)	Peak (%)	Mean (%)
S1	60.6	55.8	62.3	54.9	S11	53.3	49.0	56.8	52.6
S2	78.1	70.2	72.4	67.6	S12	96.4	88.6	96.0	87.5
S3	91.7	81.8	88.3	78.8	S13	60.6	56.8	63.5	58.1
S4	59.7	52.8	55.9	50.0	S14	86.9	78.9	88.0	78.7
S5	61.9	55.0	62.5	53.5	S15	98.9	94.2	99.4	94.3
S6	61.7	55.6	61.8	55.2	S16	70.0	60.0	71.6	66.1
S7	55.6	51.5	56.6	50.8	S17	85.6	77.2	86.3	78.4
S8	79.7	74.2	76.8	70.5	S18	91.7	82.6	91.8	83.2
S9	85.3	72.6	82.7	70.5	S19	89.4	78.3	88.2	77.7
S10	84.7	76.3	83.5	75.7	S20	67.5	61.0	77.2	69.6
Average	71.9	64.6	70.3	62.8		80.0	72.7	81.9	74.6

## Discussion

In this paper, we compared the performance of a supervised retraining approach with a semi-supervised retraining approach for ERD/S-based co-adaptive BCI systems. In two groups consisting of 10 participants each, we evaluated each approach online, while we simulated its counter model offline. We evaluated the accuracy for 180 feedback trials per participant. In both approaches, the grand average for online and offline analysis was significantly higher than chance with 71.9% for group A and 80% for group B for peak accuracies and 64.6% respective 72.7% for the mean over the feedback period. When directly comparing both approaches within the groups (where one is always simulated offline), we could show that between both approaches no significant difference, neither in peak nor mean accuracy, could be found. However, on grand average, the supervised model yielded better classification accuracies of about 2%. Nevertheless, we can surmise the alternative hypothesis, namely that the semi-supervised approach does not perform significantly worse than the supervised approach, can be accepted.

### Performance evaluation

In this study, we deliberately extended the duration of our experiment not only by recording a considerably higher number of trials than in our previous co-adaptive attempts [20, 32, 42], but also by including extended breaks. In this way, we created alternating stages of BCI control and stages where no action was needed. Our results show that, despite the extended duration of the experiment and the breaks, no significant drop in performance between stages could be determined for both supervised and semi-supervised approaches. This is an additional advantage of co-adaptive BCI systems for long-term use. Moreover, it shows that the performance of the semi-supervised approach is not significantly affected by the lack of true labeling of incoming data or the extended time of use.

The number of retrainings for the semi-supervised approach decreased in comparison to the supervised approach (on average 11 vs. 27 retrainings). This was due to an additional selection criterion that excluded trials in which peak accuracies were within the first and fourth quartile of all available data for retraining: low class probabilities therefore indicated that they were close to the LDA decision boundary (e.g., within the first (below 25%) quartile) and that the classifier is more uncertain. On the contrary, high class probabilities indicated that they might not only be very distant to the LDA decision boundary but also distant to the classes’ own mean distribution (e.g., within the fourth (above 75%) quartile), making them outliers. Our results indicate that despite having a reduced number of retrainings, performance was not significantly affected. In other words, it is possible to retain all positive aspects that come with the co-adaptive approach such as shortened period where no feedback was provided or keeping the user engaged, while using only a fraction of labeled data necessary.

In direct comparison to our preceding co-adaptive work [20], lower accuracies were achieved in group A and almost equal peak and mean results in group B regardless of whether evaluated offline or online.

Both groups contained participants who scored more than 90% in online peak accuracy which indicates a considerable level of control. Only one subject in the online experiment did not achieve a classification performance above chance level. A direct comparison with other methods other than [19, 20] is difficult due to differences in paradigms, mental tasks, and/or experiment duration. Nevertheless, our results are within the same range as shown by the co-adaptive assessments of Acqualagna et al. in a large population study (*n* = 168, two centers, mean 72.4 and 78.3% accuracy) [43].

In comparison to the work in unsupervised adaptation of Hasan and Gan [21] and Gan et al. [22], we could show that our approach of using semi-supervised learning to adapt the classifier without true class labels works also in an online setting over an extended period of time—without a significant decrease in performance compared to the supervised adaptation. Moreover, our method of selecting classifier output labels for retrainings in an unsupervised way is relatively simple and less costly (in terms of computational load) compared to the more complex mathematical methods of unsupervised updates described in [21, 23], and [22].

Of particular interest is the work from Müller et al. [15] who tackled the two-learners problem (Human ↔ Machine) in a theoretical co-adaptive system simulation. In this simulation, the authors focused on the bidirectional communication between human and machine. They show that the error rates of non-adaptive BCI systems stagnate if the machine’s learning rate is zero, which is not the case for co-adaptive BCIs. Additionally, they show that best results can be achieved by choosing controlled (mid range) learning rates for the machine, a concept which is similar to our semi-supervised approach. For retraining CSP and classification models, we only incorporated “mid-range” trials with respect to their class probabilities.

### Power modulations

We also investigated the underlying power modulations for each participant by calculating the PSD estimate for each condition. In most participants, distinct power modulations can be found, most exemplary in, e.g., S12 and S19. A decrease in power for the hand MI condition contralateral in C3 can be found (e.g., S12), while distinct power decrease for condition feet MI can be seen centrally at Cz (e.g., S19). The PSDs also show that for two participants (S10, S11), especially S10, data was contaminated with movement artifacts, most likely induced by head movements. One must therefore take this point into consideration when interpreting these participant’s results since this effect could have partially contributed to their classification performance.

### Limitations and applicability for future long-term use BCIs

In this study, we performed a between-group analysis (*n* = 10) for comparing both investigated approaches. This means that each participant only performed one approach online, while we simulated the other offline. We deliberately accepted this trade-off to avoid training effects as well as bias toward the experiment which might have influenced participant performance.

We successfully combined a co-adaptive BCI with a semi-supervised recurrent retrainer unit. We could show that this novel approach is not only feasible but also works with no significant performance loss in comparison to supervised analogues [20]. We believe that this approach can be further exploited for true self-paced, long-term BCI use, where instructions are not predetermined, rather than causally determined by the BCI user. Using already executed BCI commands as “anchor” points, new trials could be extracted during runtime and added to the datapool of the retraining unit, where a class label could be artificially generated for retraining the BCI.

To further enhance reliability and stability of the BCI, it would be advisable to detect and deal with artifactual data already on site within the online BCI model rather than on the recurrent retraining unit. This could not only reduce the number of false-positive detections due to artifacts but also deny inclusion into the retraining data pool. Previous studies have already shown the benefit of detecting and dealing with artifactual EEG online [44–48].

Future investigations will show whether this approach can be scaled up by incorporating additional mental imagery classes or a rest class. Additionally, it would also be interesting to apply this concept on detection and classification of low-frequency components triggered by complex movements such as described in [49–53].

## Conclusion

In this paper, we compared a supervised with a semi-supervised retraining approach for ERD-based co-adaptive BCI systems. We could show that despite the low number of labeled data (only 40 TPC of a total of 190 TPC), the semi-supervised approach yielded similar performance results when compared to the supervised approach. These findings may contribute to developing BCIs for long-term use, in which continuous adaptation becomes imperative for maintaining a meaningful BCI performance.

## Electronic supplementary material

Fig. S1 (PNG 52 kb)

Fig. S2 (PNG 75 kb)
