# Supplementary figures and images for: Direct comparison of supervised and semi-supervised retraining approaches for co-adaptive BCIs
Source: Med Biol Eng Comput. 2019 Sep 14;57(11):2347–57. doi: 10.1007/s11517-019-02047-1 (PMC6828633; doi:10.1007/s11517-019-02047-1)

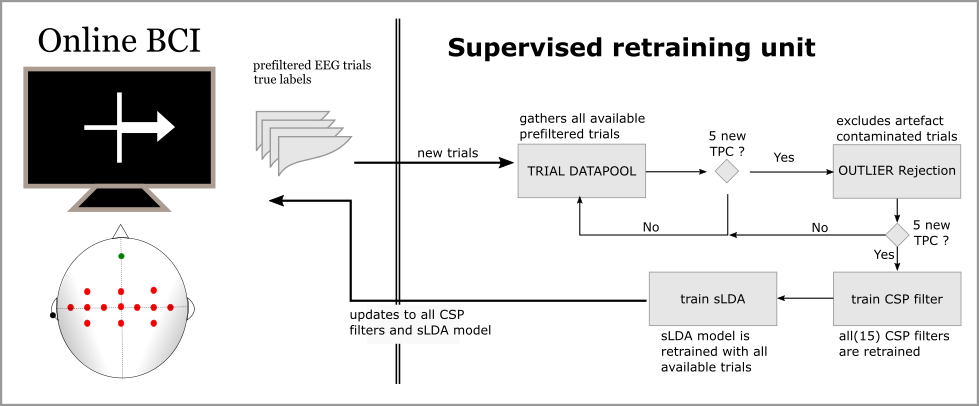

Supplement: Supplementary file 1 — (PNG 52 kb) [file 11517_2019_2047_MOESM1_ESM.png]

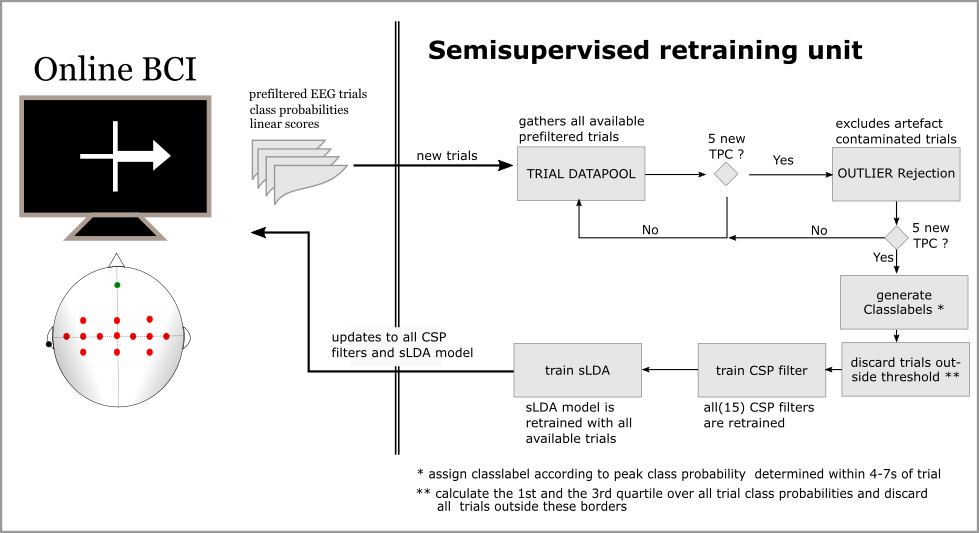

Supplement: Supplementary file 2 — (PNG 75 kb) [file 11517_2019_2047_MOESM2_ESM.png]
